# Chromosomal localization of cohesin is differentially regulated by WIZ, WAPL, and G9a

**DOI:** 10.1186/s12864-022-08574-w

**Published:** 2022-04-30

**Authors:** Megan Justice, Audra F. Bryan, Juanita C. Limas, Jeanette Gowen Cook, Jill M. Dowen

**Affiliations:** 1 Integrative Program for Biological and Genome Sciences, University of North Carolina, Chapel Hill, NC USA; 2 Curriculum in Genetics and Molecular Biology, University of North Carolina, Chapel Hill, NC USA; 3 Department of Pharmacology, University of North Carolina, Chapel Hill, NC USA; 4 Department of Biochemistry and Biophysics, University of North Carolina, Chapel Hill, NC USA; 5 Lineberger Comprehensive Cancer Center, University of North Carolina at Chapel Hill, Chapel Hill, NC USA; 6 Department of Biology, University of North Carolina, Chapel Hill, NC USA

**Keywords:** Cohesin, Chromosome, Structure, Genome, Organization, Chromatin, Localization, WIZ

## Abstract

**Background:**

The cohesin complex is essential for proper chromosome structure and gene expression. Defects in cohesin subunits and regulators cause changes in cohesin complex dynamics and thereby alter three-dimensional genome organization. However, the molecular mechanisms that drive cohesin localization and function remain poorly understood.

**Results:**

In this study, we observe that loss of WIZ causes changes to cohesin localization that are distinct from loss of the known WIZ binding partner G9a. Whereas loss of WIZ uniformly increases cohesin levels on chromatin at known binding sites and leads to new, ectopic cohesin binding sites, loss of G9a does not. Ectopic cohesin binding on chromatin after the loss of WIZ occurs at regions that are enriched for activating histone modifications and transcription factors motifs. Furthermore, loss of WIZ causes changes in cohesin localization that are distinct from those observed by loss of WAPL, the canonical cohesin unloading factor.

**Conclusions:**

The evidence presented here suggests that WIZ can function independently from its previously identified role with G9a and GLP in heterochromatin formation. Furthermore, while WIZ limits the levels and localization pattern of cohesin across the genome, it appears to function independently of WAPL-mediated cohesin unloading.

**Supplementary Information:**

The online version contains supplementary material available at 10.1186/s12864-022-08574-w.

## Background

The cohesin complex plays important roles in the regulation of gene expression, genome organization, DNA replication, DNA repair, and sister chromatid cohesion [1, 2]. Knowledge of how cohesin performs these varied functions at specific sites across the genome is limited. The ring-shaped cohesin complex is thought to be loaded onto DNA at active enhancers and promoters, translocate along or extrude DNA, and then unload at sites distal to the loading sites [3]. NIPBL (Nipped B-Like) regulates the level of cohesin on the genome by facilitating the interaction of cohesin and DNA, and by stimulating the ATP hydrolysis activity of the cohesin subunits SMC1 and SMC3 during DNA extrusion [4–7]. Removal of cohesin from the genome occurs in one of two ways: 1) At the onset of anaphase, the RAD21 subunit of cohesin is cleaved by the enzyme Separase to allow for separation of sister chromatids in mitosis [8]. 2) Rather than cleaving a cohesin subunit, WAPL (Wings Apart-Like) can remove cohesin from chromatin throughout the cell cycle by opening a “DNA exit gate” in the complex, rather than cleavage of a subunit [9–14]. Depletion of WAPL results in increased levels of cohesin on the genome [10, 15]. Many questions still remain regarding the molecular details of cohesin occupancy on the genome, including whether additional cohesin regulators remain to be identified and characterized.

Recently, WIZ (Widely Interspaced Zinc fingers protein) was identified as a binding partner of the cohesin complex [16]. The WIZ protein is ubiquitously expressed in nearly all cell types and contains six C2H2-type zinc finger domains, which are unusually widely spaced compared to other zinc finger-containing proteins [16, 17]. The most well-studied role of WIZ is in the stabilization of the G9a/GLP histone lysine methyltransferase complex on chromatin [17, 18]. In this complex, WIZ recruits the methyltransferase enzymes to DNA leading to mono- and di-methylation of H3K9 across the genome. Until recently, the role of WIZ in gene regulation was thought to occur solely through its known role in mediating heterochromatin formation. However, it is now known that WIZ can also function in complex with cohesin and CTCF. WIZ physically interacts with cohesin and CTCF at the anchors of DNA loops that control gene expression and cellular identity [16].

Quantitative ChIP-seq for the cohesin subunit RAD21 following loss of WIZ in mouse embryonic stem cells (mESCs) revealed a genome-wide increase in cohesin occupancy on chromatin, as measured in two distinct analyses [16]. First, peak-calling and overlap identified 25,000 ectopic cohesin peaks in the absence of WIZ [16]. While cohesin peaks that are shared between WT and *Wiz*^*del*^ cells frequently overlap CTCF sites and DNA loop anchors, the ectopic cohesin peaks rarely overlap these elements and are instead largely intergenic [16]. Second, quantitative analysis of changes in ChIP-seq signal at retained cohesin sites upon the loss of WIZ revealed a significant increase in signal at nearly all retained cohesin peaks [16]. These data revealed that WIZ can also act as a regulator of cohesin occupancy on chromatin, though the molecular details of this relationship remain unclear.

Here, we investigate the potential molecular causes of the increased cohesin occupancy observed across the genome in the absence of WIZ. Comparison of the localization of cohesin on chromatin upon loss of WIZ, or loss of the cohesin unloader WAPL, reveals that WIZ and WAPL regulate cohesin localization in distinct ways, with loss of WIZ affecting a greater number of cohesin binding sites on chromatin. Additionally, loss of WIZ alters cohesin localization more dramatically than loss of G9a. We also identify transcription factors whose functions at ectopic cohesin binding sites may underlie the altered localization of cohesin in the absence of WIZ. Taken together, these data suggest that WIZ is an important regulator of cohesin localization on chromatin, that operates distinctly from WAPL-mediated unloading of cohesin, as well as from the role WIZ can play with G9a in histone modifying complexes.

## Results

### WIZ regulates cohesin localization in a manner distinct from the cohesin unloading factor WAPL

**Fig. 1:**
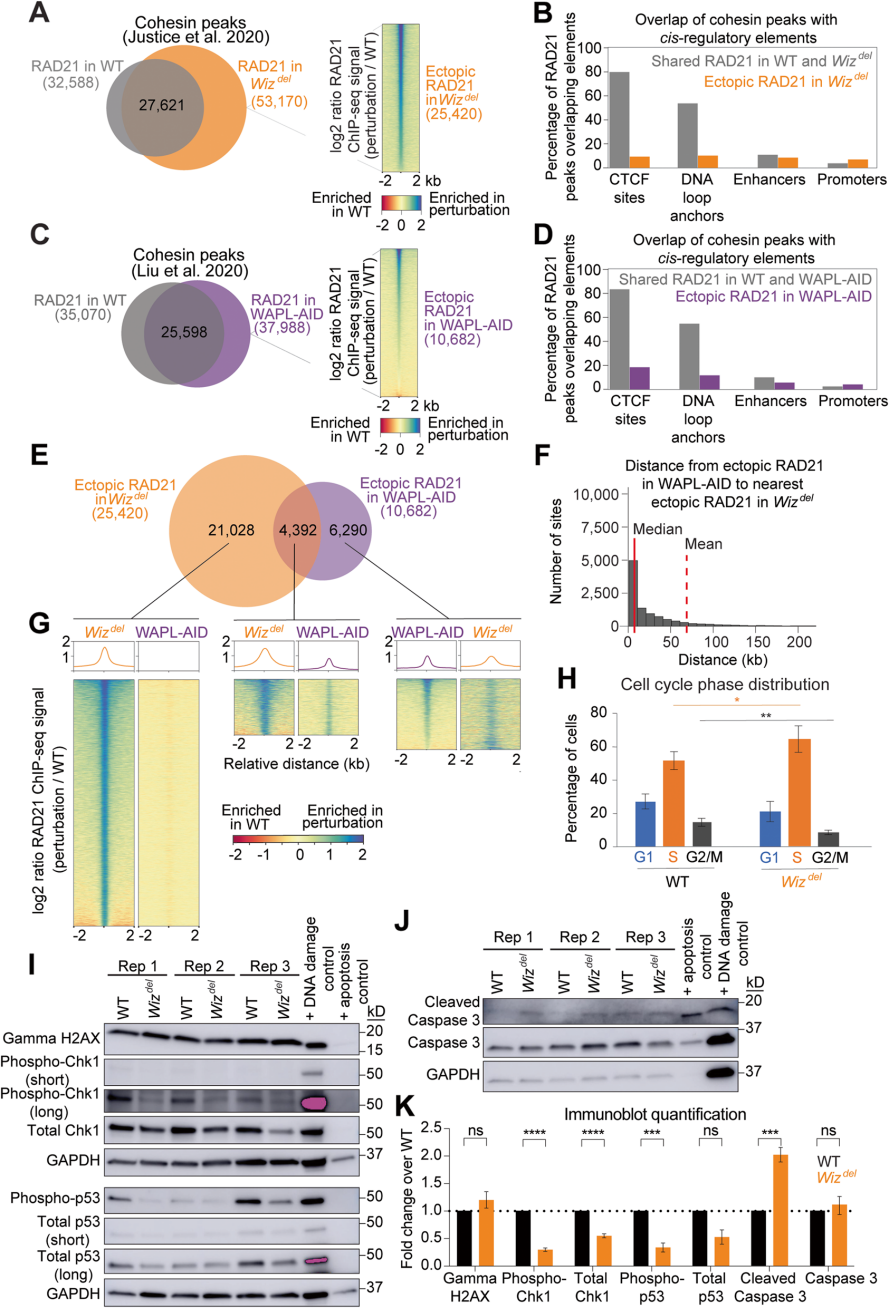
WIZ and WAPL differentially regulate cohesin localization. **A** Overlap of RAD21 ChIP-seq peaks in WT and *Wiz*^*del*^ mESCs. Heatmap shows the log2 ratio of ChIP-seq signal in *Wiz*^*del*^ vs WT. Peaks identified in *Wiz*^*del*^ cells but not WT cells, which displayed a negative log2 ratio of ChIP-seq signal were removed from further analysis. **B** Bar graph showing overlap of shared (grey) and ectopic (orange) RAD21 peaks in *Wiz*^*del*^ cells with CTCF sites, DNA loop anchors, enhancers, and promoters. **C** Overlap of RAD21 ChIP-seq peaks in WT and WAPL-AID mESCs. Heatmap shows the log2 ratio of ChIP-seq signal in WAPL-AID / WT. Peaks identified in WAPL-AID cells but not WT cells, which displayed a negative log2 ratio of ChIP-seq signal were removed from further analysis. **D** Overlap of shared (grey) and ectopic (purple) RAD21 peaks in WAPL-AID cells with CTCF sites, DNA loop anchors, enhancers, and promoters. **E** Overlap of ectopic RAD21 peaks in *Wiz*^*del*^ cells and ectopic RAD21 peaks WAPL-AID cells. Overlap is defined as within 2 kb. **F** Histogram showing the distance from an ectopic RAD21 peak in WAPL-AID cells to the nearest ectopic RAD21 peak in *Wiz*^*del*^ cells. Median (red solid line) and mean (red dashed line) of the distribution are indicated. **G** Heatmaps showing the log2 ratio of RAD21 signal (perturbation / WT) at ectopic RAD21 peaks identified in *Wiz*^*del*^ cells only (left heatmaps, sorted from highest to lowest signal in *Wiz*^*del*^ column), peaks identified as ectopic in both *Wiz*^*del*^ cells and WAPL-AID cells (center heatmaps, sorted from highest to lowest signal in *Wiz*^*del*^ column), and peaks identified only in WAPL-AID cells (right heatmaps, sorted from highest to lowest signal in WAPL-AID column). **H** Bar graph showing the percent of cells in each indicated cell cycle phase for WT and *Wiz*^*del*^ cells. Data represent *n* = 5 (WT) and *n* = 6 (*Wiz*^*del*^ cells) replicates. Asterisks represent *p* < 0.01(*) and *p* < 0.001 (**) measured using an unpaired t-test. **I** Immunoblots for indicators of apoptosis and markers of the DNA damage response in WT and *Wiz*^*del*^ whole cell extracts. Positive controls for DNA damage and apoptosis were generated as described in Methods; GAPDH serves as a loading control for each membrane (top and bottom set of blots). Purple indicates saturated signal. **J** Immunoblots for indicators of apoptosis in WT and *Wiz*^*del*^ whole cell extracts. GAPDH serves as a loading control. **K** Quantification of immunoblots in panels I-J and Fig. S1G-I as fold-change relative to WT after normalization to protein loading controls as described in Methods. Data are presented as mean ± SEM, *n* = 3 biological replicates. Technical replicates, where additional analysis of existing lysates was performed with additional gels and immunoblotting: Gamma H2AX *n* = 3, Phospho-Chk1 *n* = 2, Total Chk1 *n* = 2, Phospho-p53 *n* = 2, Total p53 *n* = 1, Cleaved Caspase 3 *n* = 2, and Caspase 3 *n* = 2. Paired, two-tailed t-tests were performed to determine the statistical significance of differences in protein levels between WT and *Wiz*^*del*^ cells. *P < 0.05, **P < 0.01, ***P < 0.001, ****P < 0.0001

WAPL was termed a cohesin unloader after the loss of WAPL was shown to increase the total levels of cohesin on chromatin [10, 12, 19]. To determine whether WIZ functions with, or in a manner similar to WAPL, in regulating the genomic localization of cohesin, analysis of RAD21 (a core cohesin complex member) ChIP-seq data was performed in mESCs lacking WAPL (WAPL-AID cells) or lacking WIZ (*Wiz*^*del*^ cells) [16] (Table S1). Since WAPL is an essential gene, depletion of WAPL was achieved with an auxin inducible degradation system, WAPL-AID . *Wiz*^*del*^ cells have a homozygous deletion in the *Wiz* gene that leads to a lack of WIZ protein, yet the cells display normal steady state levels of cohesin subunits [16]. Following loss of WIZ, the total number of RAD21 peaks identified increased compared to WT cells (~ 53,000 vs. ~ 32,500) (Fig. 1A). Analysis of spike-in normalized RAD21 ChIP-seq data in *Wiz*^*del*^ cells identified 25,420 ectopic cohesin peaks that were not present in wildtype (WT) mESCs and which displayed a positive log2 ratio of ChIP-seq signal (*Wiz*^*del*^ / WT). In contrast, fewer than 5000 RAD21 peaks identified in WT are lost, or orphaned, upon loss of WIZ. Whereas RAD21 peaks shared between *Wiz*^*del*^ and WT mESCs frequently overlap CTCF sites and DNA loop anchors, the ectopic RAD21 peaks that appear in the absence of WIZ do not (Fig. 1B). Both ectopic RAD21 peaks and shared RAD21 peaks show a similar overlap with enhancers and promoters. Following WAPL degradation, the total number of RAD21 peaks identified is similar to the total number identified in WT cells (~ 38,000 vs. ~ 35,000) (Fig. 1C). While more than two-thirds of the RAD21 peaks are shared between WT and WAPL-AID cells, roughly 10,000 RAD21 peaks are lost, or orphaned, following WAPL degradation and over 10,000 ectopic RAD21 peaks with a positive log2 ratio or RAD21 ChIP-seq signal (WAPL-AID / WT) are gained. The distribution of ectopic RAD21 peaks at *cis*-regulatory elements upon loss of WAPL is similar to that of ectopic RAD21 peaks in the absence of WIZ, with the greatest difference occurring at CTCF sites and DNA loop anchors (Fig. 1D).

A direct comparison of the location of cohesin peaks revealed substantial overlap between the RAD21 peaks identified in the WT cells used in the WIZ study and WAPL study (Fig. S1A) [16]. There was also substantial overlap of the RAD21 peaks shared between WT and *Wiz*^*del*^ cells, as well as between WT and WAPL-AID cells (Fig. S1B). In contrast, there is remarkably little overlap between ectopic RAD21 peaks in *Wiz*^*del*^ cells and ectopic RAD21 peaks in WAPL-AID cells, even when overlap is liberally defined as peaks within 2 kb of one another (Fig. 1E). Consistent with this finding, the mean distance from an ectopic cohesin peak in WAPL-AID cells to an ectopic cohesin peak in *Wiz*^*del*^ cells is over 68 kb, while the mean distance between shared peaks in each condition is ~ 7 kb (Figs. 1F, S1C). Furthermore, the median distance between a shared cohesin peak in WAPL-AID cells to a shared peak in *Wiz*^*del*^ cells is 0 bp due to peak overlap, while the median distance between ectopic cohesin peaks is over 7 kb (Figs. 1F, S1C). These results suggest that WAPL and WIZ do not function together in the regulation of cohesin localization, but rather function in distinct manners or at distinct locations on the genome. To determine how ectopic cohesin peaks identified in one condition are affected in the other condition, the log2 ratio (perturbation / WT) of ChIP-seq signal was determined at ectopic cohesin peaks identified in 1) only *Wiz*^*del*^ cells, 2) only WAPL-AID cells, and 3) shared by *Wiz*^*del*^ cells and WAPL-AID cells (Fig. 1G). Whereas loss of WIZ caused a strong increase in RAD21 signal at the ~ 21,000 ectopic cohesin peaks specific to *Wiz*^*de*l^ cells, WAPL depletion had no effect on RAD21 signal at these sites. The ~ 4300 ectopic RAD21 peaks identified in both *Wiz*^*de*l^ cells and WAPL-AID cells showed stronger signal in both perturbation conditions compared to their matched WT conditions. The 6200 RAD21 peaks identified as ectopic in WAPL-AID cells (compared to WT) displayed increased signal in both *Wiz*^*de*l^ cells and WAPL-AID cells, compared to their matched WT control cells. Therefore, we conclude that WIZ plays a stronger role in regulating the genomic distribution of cohesin than WAPL and that WIZ acts in a manner mostly distinct from WAPL in regulating cohesin.

A previous study found that WAPL-AID cells showed no major defects in proliferation or altered distribution across phases of the cell cycle [20]. To examine whether loss of WIZ caused a similar or different phenotype, we performed flow cytometry analysis (Fig. S1D). A significant increase in the percent of *Wiz*^*del*^ cells in S phase was detected compared to WT cells, consistent with previous reports (Fig. 1H) [21, 22]. A significant decrease in the percentage of cells in G2/M phase was also observed in *Wiz*^*del*^ cells. To investigate whether the population doubling time of *Wiz*^*del*^ cells is altered compared to WT cells, we performed a proliferation assay (Fig. S1E). Cells were plated at a known density and counted at 24-, 52-, 64-, and 72-h post-seeding. As early as 64 h post-seeding, the relative cell number of *Wiz*^*del*^ cells was significantly decreased compared to WT. The 52- and 64-h timepoint data were used to calculate the population doubling times. The total population doubling time of *Wiz*^*del*^ cells was slightly longer than that of WT cells (~ 14 h vs ~ 13 h). Combining data from flow cytometry analysis and the population doubling times, the length for each cell cycle phase was calculated in WT and *Wiz*^*del*^ cells (Fig. S1F). These analyses revealed a significant increase in the length of S phase and a significant decrease in the length of G2/M phase in *Wiz*^*del*^ cells compared to WT. Importantly, the cell cycle changes observed in *Wiz*^*del*^ cells are likely not due to increases in activation of the S phase checkpoint or the DNA damage response, as the levels of gamma H2AX, total and phosphorylated Chk1, and total and phosphorylated p53 were not increased in *Wiz*^*del*^ cells (Fig. 1I, IK, Fig. S1G-J, Figs. S2-S3). In fact, significant decreases in total and phosphorylated Chk1, as well as phosphorylated p53, were observed. It is not clear why *Wiz*^*del*^ cells have reduced levels of the cell stress indicators phospho-Chk1 and phospho-p53, but it could be related to decreased expression or stability of Chk1 or p53, or disregulation of cellular state. *Wiz*^*del*^ cells did display a 2-fold increase in cleaved Caspase 3, a marker of apoptosis, yet flow cytometry data showed a similar level of sub-G1 cells in both *Wiz*^*del*^ and WT cell lines (< 2%) (Fig. 1D, J-K, Fig. S1I, Figs. S2-S3). Our results indicate that loss of WIZ alters the cell cycle, while a previous report suggests that loss of WAPL does not, however since these findings were made in different studies it is possible that technical differences underlie this interpretation. Together, these ChIP-seq and flow cytometry data suggest that *Wiz*^*del*^ cells exhibit an altered cell cycle profile and increased cohesin binding, which are phenotypes not overlapping with loss of WAPL.

### WIZ regulates cohesin localization independently of G9a

**Fig. 2:**
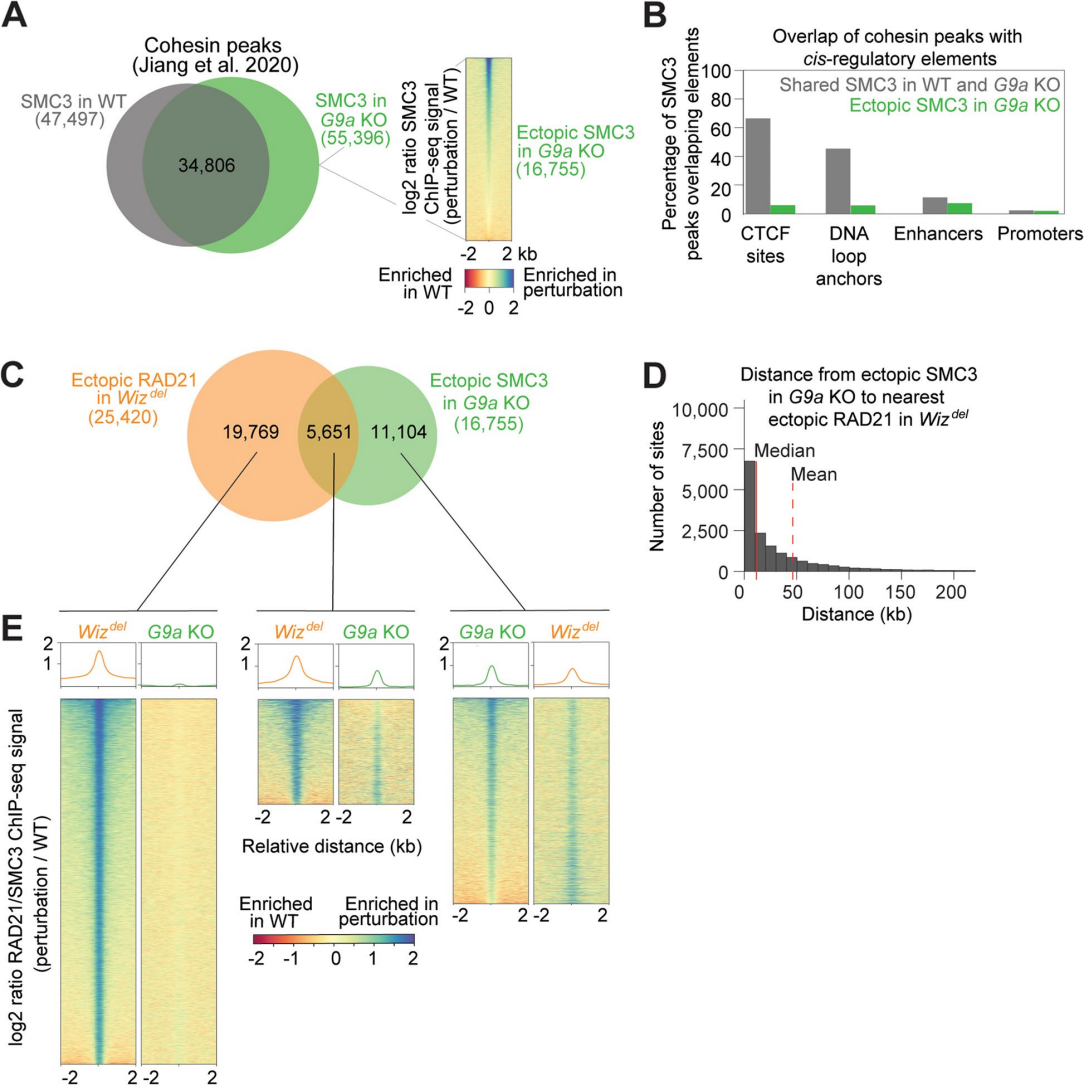
WIZ regulates cohesin localization independently of G9a. **A** Overlap of SMC3 ChIP-seq peaks in WT and *G9a* KO cells. Heatmap shows the log2 ratio of ChIP-seq signal in *G9a* KO vs WT. Peaks identified in *G9a* KO cells but not WT cells which displayed a negative log2 ratio of ChIP-seq signal were removed from further analysis. **B** Overlap of shared (grey) and ectopic (green) SMC3 peaks with CTCF sites, DNA loop anchors, enhancers, and promoters. **C** Overlap of ectopic RAD21 peaks in *Wiz*^*del*^ cells with ectopic SMC3 peaks in *G9a* KO cells. Overlap is defined as within 2 kb. **D** Histogram showing the distance from an ectopic SMC3 peak in *G9a* KO cells to the nearest ectopic RAD21 peak in *Wiz*^*del*^ cells. Median (red solid line) and mean (red dashed line) of the distribution are indicated. **E** Heatmaps showing the log2 ratio of cohesin signal (perturbation / WT) at ectopic cohesin peaks identified in *Wiz*^*del*^ cells only (left heatmaps, sorted from highest to lowest signal in *Wiz*^*del*^ column), peaks identified as ectopic in both *Wiz*^*del*^ cells and *G9a* KO cells (center heatmaps, sorted from highest to lowest signal in *Wiz*^*del*^ column), and peaks identified only in *G9a* KO cells (right heatmaps, sorted from highest to lowest signal in *G9a* KO column)

The most well-studied function of WIZ is in the stabilization of G9a/GLP histone lysine methyl-transferase complex on chromatin. This complex, made up of WIZ, G9a, GLP and ZNF644, facilitates deposition of the repressive histone modifications H3K9me1 and H3K9me2 [18, 23]. siRNA knockdown of WIZ results in a decrease in total G9a protein levels in the cell, as well as decreases in G9a, GLP, and H3K9me2 levels on chromatin [18]. Since cohesin loading preferentially occurs at nucleosome-depleted regions, it is possible that increased cohesin binding in the absence of WIZ is due to disruption of G9a-mediated heterochromatin [24, 25]. To investigate this, we determined how loss of G9a affects cohesin localization on chromatin in comparison to loss of WIZ. A direct comparison of the location of WT RAD21 peaks from the *Wiz*^*del*^ study and WT SMC3 (a core cohesin subunit) peaks from the *G9a* KO study revealed substantial overlap in cohesin localization on chromatin in WT cells [16, 26] (Fig. S4A). Overlap of SMC3 ChIP-seq peaks from *G9a* KO and WT mESCs revealed ~ 16,000 ectopic cohesin peaks and nearly 13,000 orphaned cohesin peaks upon loss of G9a (Fig. 2A) [26]. Like ectopic cohesin peaks in *Wiz*^*del*^ and WAPL-AID cells, ectopic cohesin peaks in *G9a* KO cells show relatively little overlap with CTCF sites or DNA loop anchors (Fig. 2B). There was substantial overlap of RAD21 peaks shared between WT and *Wiz*^*del*^ cells with SMC3 peaks shared between WT and G9a KO cells (Fig. S4B). Despite possessing a similar number of ectopic cohesin peaks, *Wiz*^*del*^ cells and *G9a* KO cells display largely distinct sets of ectopic cohesin peaks (Fig. 2C). The average distance from an ectopic cohesin peak identified in *G9a* KO cells to an ectopic cohesin peak identified in *Wiz*^*del*^ cells is over 45 kb, more than double the average distance between shared cohesin peaks in each condition (Figs. 2D, S4C). Furthermore, the median distance between ectopic SMC3 peaks in *G9a* KO and ectopic RAD21 peaks in *Wiz*^*del*^ cells is ~ 11 kb, as opposed to a median of 0 bp separating shared SMC3 peaks from shared RAD21 peaks, since many of these peaks overlap (Figs. 2D, S4C). While ectopic cohesin peaks identified in *G9a* KO cells show increased cohesin signal in *Wiz*^*del*^ cells, ectopic cohesin peaks in *Wiz*^*del*^ cells do not show altered cohesin levels in *G9a* KO cells, suggesting that the role of WIZ in cohesin localization is largely independent of G9a (Fig. 2E). Furthermore, ectopic cohesin peaks identified in *G9a* KO cells show little overlap with ectopic cohesin peaks identified in WAPL-AID cells, indicating that G9a and WAPL have different effects on cohesin positioning (Fig. S4D-E). At the ~ 19,700 ectopic cohesin peaks specific to *Wiz*^*de*l^ cells, loss of G9a had no effect on SMC3 signal. The ~ 5600 ectopic cohesin peaks identified in both *Wiz*^*de*l^ cells and *G9a* KO cells showed stronger signal in both perturbation conditions compared to their matched WT conditions. The 11,100 SMC3 peaks identified as ectopic in *G9a* KO cells (compared to WT) display increased cohesin signal in *Wiz*^*de*l^ cells and *G9a* KO cells compared to their WT control cells. Taken together, these results suggest that loss of WIZ does not genocopy loss of G9a, and that WIZ causes a robust increase in cohesin binding to new sites across the genome.

### Cohesin levels are differentially regulated by WIZ, G9a and WAPL

**Fig. 3:**
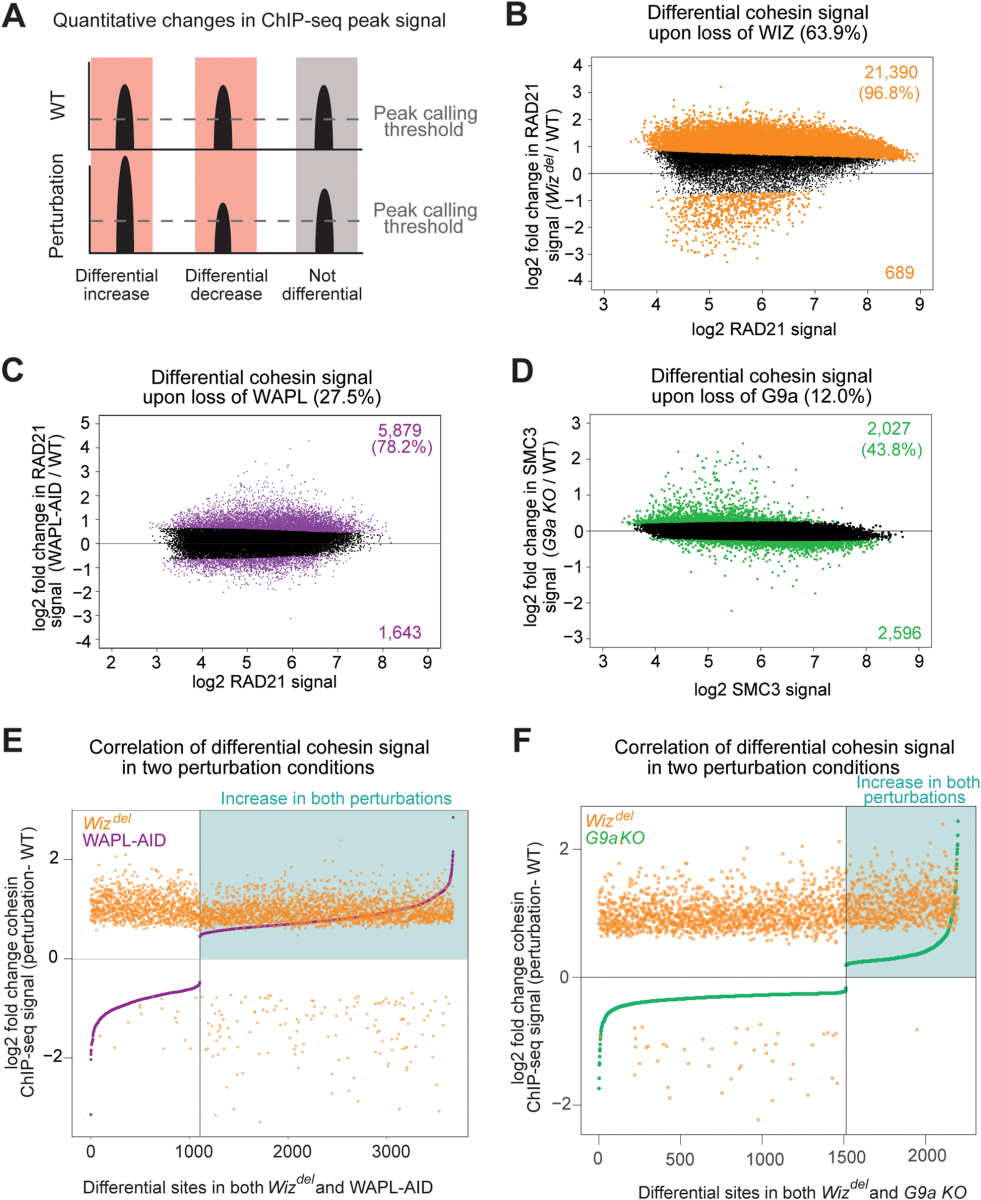
Distinct roles for WIZ, WAPL, and G9a in cohesin localization. **A** Depiction of differential occupancy analysis. ChIP-seq signal at peaks called in both WT and perturbation conditions is quantitatively compared, with sites showing significant increases or decreases in signal being called as differentially occupied. **B** Differential RAD21 signal analysis in *Wiz*^*del*^ cells. Total percentage of differentially occupied retained sites is in parentheses in the panel title. Differential sites are colored orange. (**C**) Differential RAD21 signal analysis in WAPL-AID cells. Differential sites are colored purple. **D** Differential SMC3 signal analysis in *G9a* KO cells. Differential sites are colored green. **E** Comparison of change in cohesin ChIP-seq signal at differential sites identified in both *Wiz*^*del*^ and WAPL-AID cells. The log2 fold change in cohesin signal at differential sites in *Wiz*^*del*^ cells is colored orange. The log2 fold change in cohesin signal at differential sites in WAPL-AID cells is colored purple. The sites are sorted based on the signal in WAPL-AID cells. The sites which show an increase in ChIP-seq signal in both conditions appear in the teal quadrant. **F** Comparison of change in cohesin ChIP-seq signal at differential sites in both *Wiz*^*del*^ and *G9a* KO cells. The log2 fold change in RAD21 signal at each site in *Wiz*^*del*^ cells is colored orange. The log2 fold change in SMC3 signal at each site in *G9a* KO cells is colored green. The sites are sorted based on the signal in G9a KO cells. The sites which show an increase in ChIP-seq signal in both conditions appear in the teal quadrant

In addition to the identification of shared or distinct cohesin peaks, we performed a quantitative analysis of reads at retained cohesin binding sites to identify significant quantitative changes in cohesin levels at retained cohesin binding sites in cells lacking WIZ, WAPL, and G9a (Fig. 3A). Loss of WIZ results in differential cohesin levels at 64% of retained binding sites (Fig. 3B). Furthermore, nearly all the differential sites (96.8%) displayed increased cohesin signal in *Wiz*^*del*^ cells compared to WT. In contrast, analysis of differential cohesin levels in WAPL-AID cells revealed a moderate effect on cohesin ChIP-seq signal at retained sites (27.5% differential) (Fig. 3C). Consistent with its canonical role as cohesin unloader, loss of WAPL led to increased cohesin signal at 78% of differential sites. Loss of G9a had the smallest effect on cohesin levels out of the conditions examined, with only 12% of retained sites displaying significant changes in cohesin ChIP-seq signal (Fig. 3D). Additionally, only ~ 44% of these differential cohesin sites displayed increased signal in the *G9a* KO cells, suggesting that loss of G9a has a variable effect on cohesin levels while loss of WAPL or WIZ causes a strong and preferential increase in cohesin levels on chromatin.

Comparison of differentially occupied sites in each condition (colored dots in Fig. 3B and C) revealed nearly 3500 cohesin sites which are differentially occupied in both WAPL-AID cells versus control cells and differentially occupied in *Wiz*^*del*^ cells versus control cells (Fig. 3E). While many of these sites display increased cohesin signal in both conditions (teal box), roughly 1000 sites are oppositely affected by the two perturbations (top left or bottom right quadrants). A comparison of differentially occupied sites in the absence of WIZ or G9a (colored dots in Fig. 3B and D) revealed that while 2500 sites display altered cohesin occupancy upon loss of WIZ or G9a, most of these sites are oppositely regulated by the perturbations (Fig. 3F). Nearly all the sites similarly regulated by WIZ and G9a display lower cohesin signal in *G9a* KO cells than *Wiz*^*del*^ cells. Taken together, these data suggest that WIZ regulates cohesin levels at far more binding sites than WAPL or G9a, and that in the absence of WIZ there is a consistent and strong increase in cohesin signal at many sites across the genome, while loss of WAPL causes a moderate increase in cohesin levels at some retained binding sites and loss of G9a causes relatively few changes to cohesin levels on the genome.

### Ectopic cohesin peaks in *Wiz*^*del*^ cells occur in active chromatin

**Fig. 4:**
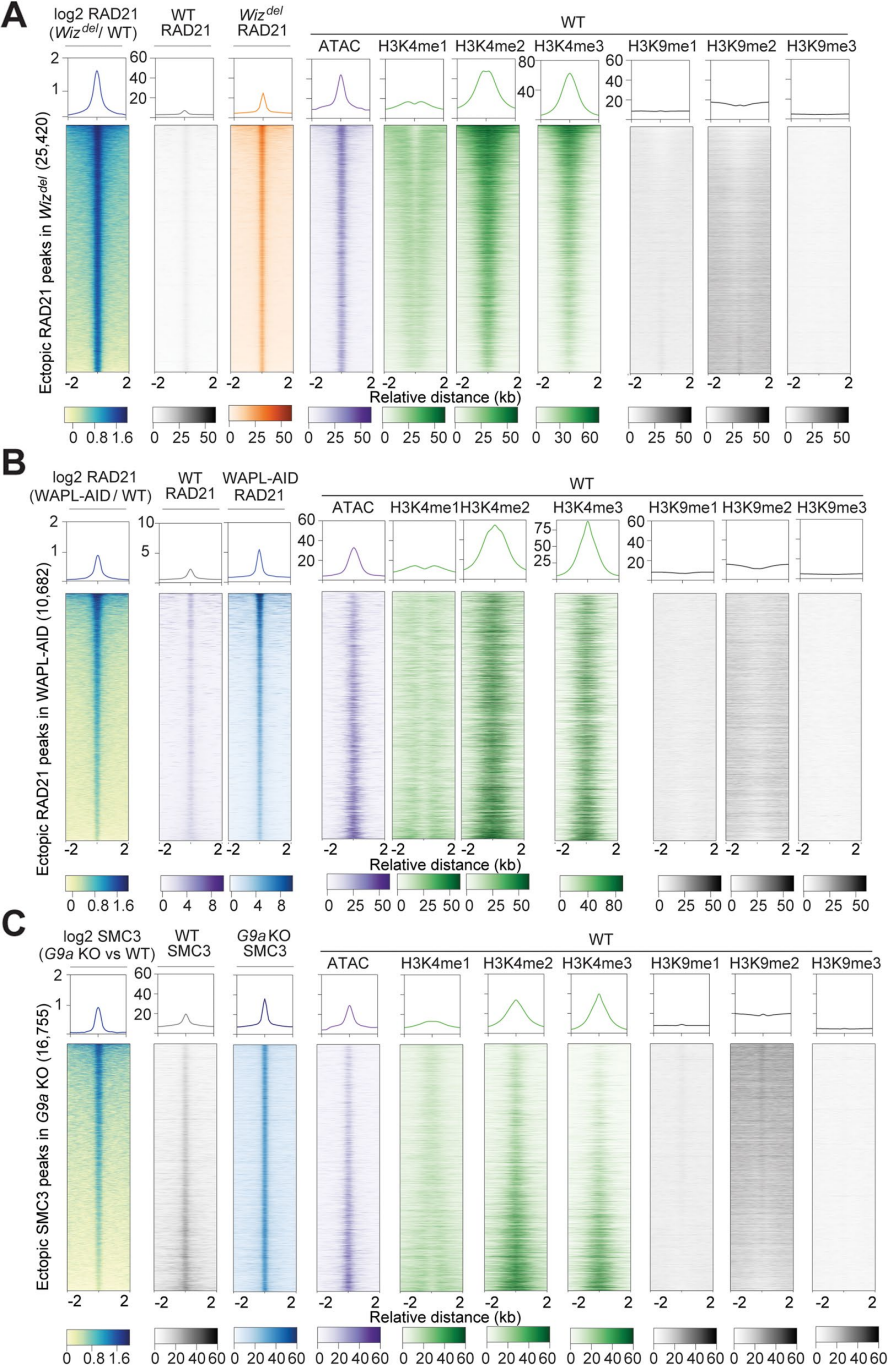
Ectopic cohesin peaks in *Wiz*^*del*^, WAPL-AID, and *G9a* KO cells display differential chromatin landscapes. **A** Heatmaps showing RAD21 ChIP-seq signal (log2 (*Wiz*^*del*^ / WT)), WT RAD21 ChIP-seq signal, *Wiz*^*del*^ RAD21 ChIP-seq signal, WT ATAC-seq signal, and WT ChIP-seq signal for H3K4me1, H3K4me2, H3K4me3, H3K9me1, H3K9me2, and H3K9me3 at ectopic RAD21 peaks detected in *Wiz*^*del*^ cells. **B** Heatmaps showing RAD21 ChIP-seq signal (log2 (WAPL-AID / WT)), WT RAD21 ChIP-seq signal, WAPL-AID RAD21 ChIP-seq signal, WT ATAC-seq signal, and WT ChIP-seq signal for H3K4me1, H3K4me2, H3K4me3, H3K9me1, H3K9me2, and H3K9me3 at ectopic RAD21 peaks detected in WAPL-AID cells. **C** Heatmaps showing SMC3 ChIP-seq signal (log2 (*G9a* KO / WT)), WT RAD21 ChIP-seq signal, *G9a* KO SMC3 ChIP-seq signal, WT ATAC-seq signal, and WT ChIP-seq signal for H3K4me1, H3K4me2, H3K4me3, H3K9me1, H3K9me2, and H3K9me3 at ectopic SMC3 peaks detected in *G9a* KO cells

**Fig. 5:**
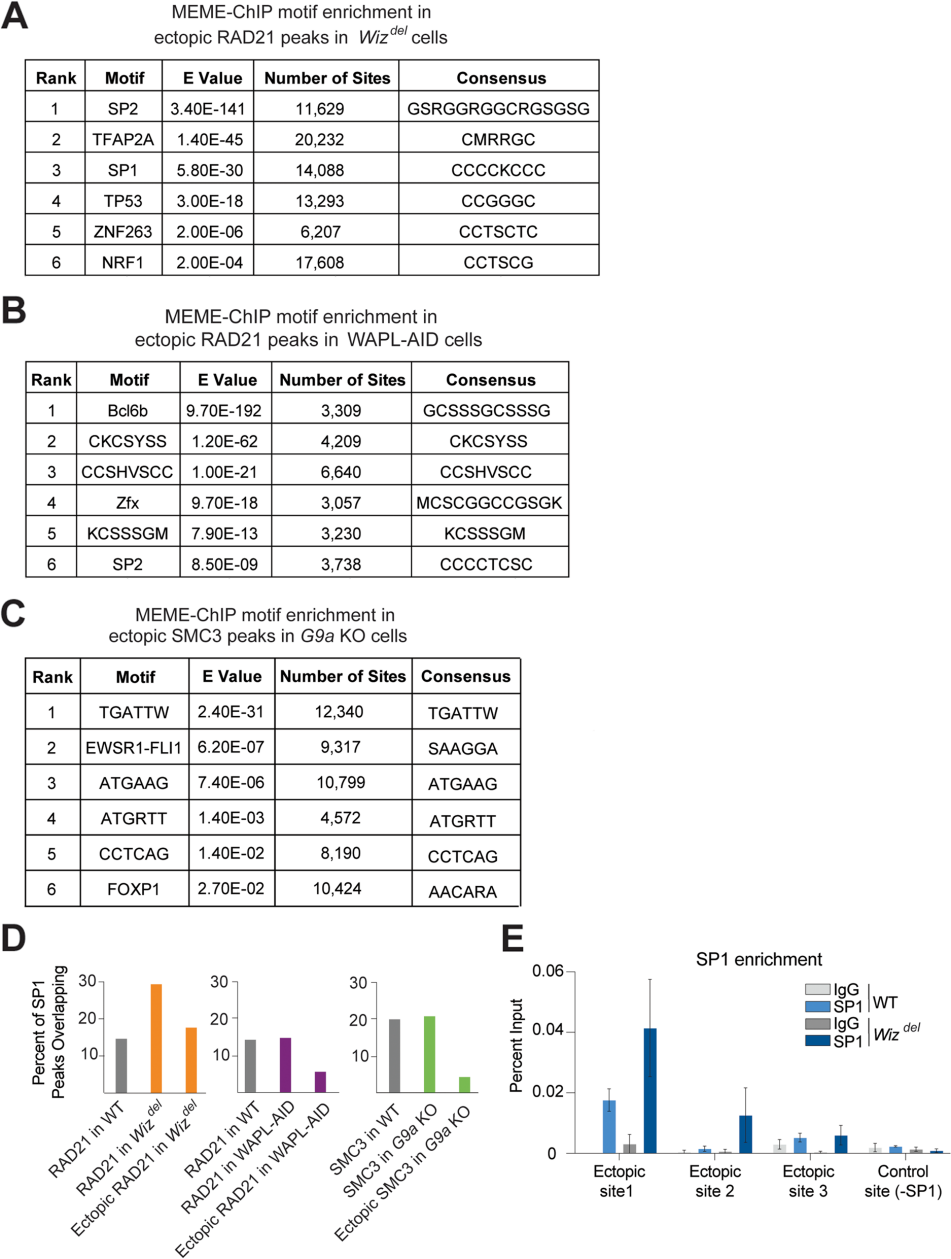
Ectopic cohesin peaks in *Wiz*^*del*^ cells are enriched for various transcription factor motifs. **A** Table showing top 6 motifs identified by MEME-ChIP motif enrichment analysis for ectopic RAD21 peaks in *Wiz*^*del*^ cells. RAD21 peaks shared between *Wiz*^*del*^ and WT cells were used as background in the analysis. **B** Table showing top 6 motifs identified by MEME-ChIP motif enrichment analysis for ectopic RAD21 peaks in WAPL-AID cells. RAD21 peaks shared between WAPL-AID and WT cells were used as background in the analysis. **C** Table showing top 6 motifs identified by MEME-ChIP motif enrichment analysis for ectopic SMC3 peaks in *G9a* KO cells. SMC3 peaks shared between *G9a* KO and WT cells were used as background in the analysis. **D** Bar graph showing the percentage of SP1 peaks in WT mESCs which overlap all cohesin (RAD21 or SMC3) peaks in WT, all cohesin (RAD21 or SMC3) peaks in the perturbation condition (*Wiz*^*del*^, WAPL-AID, or G9a), and all ectopic cohesin (RAD21 or SMC3) peaks in the perturbation condition (*Wiz*^*del*^, WAPL-AID, or G9a). **E** SP1 ChIP-qPCR was performed in WT and *Wiz*^*del*^ cells at three RAD21 ectopic sites, as well as a control site. Data are presented as mean ± SEM, *n* = 3 independent ChIP experiments performed with technical triplicates

The result that cohesin localization is differentially affected by loss of WIZ versus G9a suggests that the increased cohesin signal observed in *Wiz*^*del*^ cells is not fully explained by recruitment of cohesin to newly accessible sites due to loss of heterochromatin. To further investigate the chromatin landscape of ectopic cohesin peaks identified in each condition, we examined the enrichment of various euchromatic and heterochromatic histone post-translational modifications (PTMs), as well as chromatin accessibility via ATAC-Seq, in WT cells at the genomic locations that gain ectopic cohesin peaks in *Wiz*^*del*^ cells, WAPL-AID cells, or *G9a* KO cells. To fully appreciate the relationship between the change in cohesin signal at ectopic cohesin peaks and the enrichment of histone PTMs, we sorted each set of ectopic cohesin peaks in descending order of the log2 ratio in signal between perturbation and WT conditions. The genomic locations of ectopic cohesin peaks identified in *Wiz*^*del*^ cells are not enriched for the heterochromatic marks H3K9me1, H3K9me2, and H3K9me3 in WT cells (Fig. 4A). Rather, the loci of ectopic cohesin peaks are enriched for the euchromatic marks H3K4me2 and H3K4me3, and display robust ATAC-seq signal, suggesting that the sites that gain the most cohesin upon loss of WIZ are already accessible in WT mESCs. Genomic locations of cohesin peaks shared between WT and *Wiz*^*del*^ cells showed a similar pattern of enrichment for euchromatic and not heterochromatic histone modifications (Fig. S5A). Ectopic cohesin peaks in WAPL-AID and *G9a* KO cells also tended to be marked with euchromatic but not heterochromatic histone modifications in WT cells (Fig. 5B-C). The same pattern was true of the cohesin peaks shared between WAPL-AID and control cells, as well as G9a KO and control cells (Fig. S5B-C). However, while the strongest ectopic cohesin peaks in *Wiz*^*del*^ cells also showed the strongest signals for H3K4me1, H3K4me2, or H3K4me3 marks, the strongest ectopic cohesin peaks in WAPL-AID and *G9a* KO cells did not (Fig. 4B-C). Instead, the strongest ectopic cohesin peaks in WAPL-AID and *G9a* KO displayed the least signal for the euchromatic marks H3K4me1, H3K4me2, or H3K4me3. Taken together, these data suggest that ectopic cohesin binding upon loss of WIZ preferentially occurs at genomic sites that are accessible in WT cells.

### Enrichment of transcription factor motifs within ectopic cohesin peaks

Given that ectopic cohesin peaks in *Wiz*^*del*^ cells accumulate at sites of accessible chromatin marked by active histone PTMs, we sought to investigate how cohesin may be recruited to these sites. To identify binding motifs within ectopic cohesin peaks in *Wiz*^*del*^ cells, we performed a differential enrichment analysis using MEME-ChIP (Table S2). A previous analysis of motifs enriched at ectopic cohesin peaks in *Wiz*^*del*^ cells failed to identify motifs that were specific to the ectopic peaks and not found in the shared cohesin peaks [16]. To refine this analysis, the width of the input sequences was decreased from 500 bp to 100 bp, centered around the peak summit. Also, a differential enrichment mode of MEME-ChIP, using cohesin peaks shared between WT and *Wiz*^*del*^ cells as background, was used to identify enriched motifs unique to *Wiz*^*del*^ cells. This revised analysis identified several transcription factor motifs specific to the ectopic class, including those for SP2, TFAP2A, SP1, TP53, ZNF263, and NRF1 (Fig. 5A). The same analysis in WAPL-AID cells revealed enrichment for the transcriptional regulators Bcl6b, Zfx, and SP2 motifs in the ectopic RAD21 peaks as well as three de novo motifs (Fig. 5B). Motifs enriched at ectopic SMC3 peaks in *G9a* KO cells included the oncofusion protein EWSR1-FLI1 and transcription factor FOXP1, as well as four de novo motifs (Fig. 5C). An analysis of publicly available SP1 ChIP-seq data confirmed that the genomic sites identified as ectopic cohesin peaks in *Wiz*^*del*^ cells are enriched with SP1 binding in WT mESCs (Fig. 5D). In contrast, ectopic cohesin peaks in cells lacking WAPL or G9a less frequently overlapped SP1 binding sites than all cohesin peaks detected in either cell line. Additionally, SP1 is enriched at sites of ectopic cohesin peaks in WT and *Wiz*^*del*^ cells (Fig. 5E). These results suggest that the ectopic binding of cohesin at euchromatic regions in *Wiz*^*del*^ cells may be due to specific interactions with factors like SP1, since distinct sets of motifs were identified within the ectopic cohesin peak lists from cells lacking WIZ, WAPL, or G9a.

## Discussion

The cohesin complex participates in various biological processes that happen on the genome and are essential for cell viability. However, many questions remain about the mechanisms dictating the loading, translocation, and unloading of the complex across the genome. WIZ was previously identified as a candidate regulator of cohesin localization, as loss of WIZ protein leads to increased cohesin ChIP-seq signal genome-wide [16]. However, molecular details of how WIZ regulates cohesin and how loss of WIZ compares to loss of other known effectors of cohesin localization was not clear. Here, we compare patterns of cohesin mislocalization upon loss of WIZ, loss of a known WIZ binding partner, G9a, and loss of the canonical cohesin unloader, WAPL. The results reveal that loss of WIZ, G9a, and WAPL do not genocopy one another. Rather the loss of these three proteins leads to distinct effects on the genome-wide distribution of cohesin and known biological functions of cohesin. Interestingly, loss of WIZ has a wide-spread effect on cohesin localization, affecting more cohesin binding sites than either loss of G9a or loss of WAPL. In addition, the effect of WIZ loss is remarkably consistent across the genome, with nearly all differentially occupied sites displaying higher signal for cohesin in the absence of WIZ. Finally, sites which become ectopically bound by cohesin in each condition display somewhat unique chromatin profiles in WT mESCs. Whereas the strongest ectopic cohesin peaks upon loss of WIZ tend to have strong signal for euchromatic histone marks, the strongest ectopic cohesin peaks upon loss of G9a are not enriched for euchromatic histone marks. Ectopic cohesin peaks in WAPL depleted cells show no correlation with the strength of euchromatic histone marks. This suggests that WIZ, G9a, and WAPL all regulate the localization of the cohesin complex via different mechanisms. Importantly, the cohesin localization patterns described in this study use ChIP-seq datasets generated independently by different groups, and therefore, could be subject to technical variation in experimentation. Nevertheless, we note that cohesin peaks in WT cells were well conserved, suggesting that cohesin ChIP-seq datasets are comparable across studies. Together, these data suggest that the role of WIZ in cohesin-mediated localization is independent of its known role with binding partner G9a, and distinct from the role of the canonical cohesin unloader WAPL in cohesin localization.

Canonically, the cohesin complex is thought to be unloaded from chromatin by the protein WAPL. Depletion of WAPL has been shown, via microscopy, to increase the residence time of the cohesin complex on chromatin [10, 12, 27]. As such, we hypothesized that loss of the WIZ protein may alter cohesin localization, similar to how the depletion of WAPL leads to a defect in WAPL-mediated unloading of the complex. However, analysis of ectopically bound cohesin peaks in cells lacking WIZ and cells lacking WAPL revealed that ectopic cohesin is distributed to a distinct set of loci in each condition. Furthermore, loss of WIZ increases cohesin signal at most ectopic cohesin sites detected in cells lacking WAPL, as demonstrated by increased RAD21 ChIP-seq signal at those sites. This is due to the fact that at certain sites RAD21 signal falls below the threshold for peak calling in *Wiz*^*del*^ cells. In contrast, loci which display ectopic cohesin peaks in the absence of WIZ show no change in cohesin signal upon loss of WAPL. Quantitative analysis of cohesin ChIP-seq signal at retained cohesin binding sites in each condition revealed that loss of WIZ results in a consistent increase in cohesin levels, whereas loss of WAPL has a more varied effect on cohesin levels. In all, these data suggest that WIZ regulates cohesin localization via a mechanism distinct from WAPL. Future studies of cohesin residence time, such as measuring recovery of GFP-tagged cohesin following photobleaching, are needed to quantitate the distinct effects of WIZ and WAPL on cohesin dynamics.

Previous studies suggest that WIZ plays a crucial role in the function of the G9a/GLP histone lysine methyltransferase complex [18, 23]. This complex deposits the repressive H3K9me1 and H3K9me2 histone modifications. Since cohesin is thought to preferentially load at and localize to euchromatic regions of the genome, we investigated whether ectopic cohesin peaks in the absence of WIZ could be appearing in newly opened regions previously repressed by the G9a complex. We therefore compared the effects of G9a loss on cohesin localization to the effects of WIZ loss on cohesin localization. While loss of WIZ often leads to an increase in RAD21 ChIP-seq signal at sites of ectopic cohesin peaks detected in the absence of G9a, loss of G9a does not result in an increase in the average ChIP-seq signal of cohesin at ectopic cohesin peaks detected in cells lacking WIZ. Few cohesin binding sites are differentially occupied in cells lacking G9a, compared to > 60% of sites in cells lacking WIZ. Furthermore, sites which are differentially occupied in both conditions show mostly opposite effects on cohesin signal, with consistently higher signal upon loss of WIZ. These data suggest that WIZ regulates cohesin at a subset of binding sites independently of its canonical binding partner G9a and provide further evidence for the newly-identified role of WIZ without G9a, in the regulation of cohesin localization across the genome.

Examination of the DNA sequences underlying ectopic cohesin peaks in *Wiz*^*del*^ cells revealed enrichment for several transcription factor motifs. The transcription factors with motifs enriched within ectopic cohesin peak sequences in cells lacking WIZ have various cellular functions. Some of these transcription factor motifs have also been identified within sequences bound by the cohesin loader, NIPBL [28]. Therefore, the ectopic cohesin peaks in *Wiz*^*del*^ cells could arise from NIPBL-mediated cohesin loading at these transcription factor binding sites. Many of the motifs enriched within ectopic cohesin peak sequences in *Wiz*^*del*^ cells, including those for Tp53, SP1, and ZNF263, are not enriched in ectopic cohesin peak sequences found in cells lacking WAPL or G9a. Many of the transcription factors with motifs enriched within *Wiz*^*del*^ ectopic cohesin peak sequences are ubiquitously expressed and participate in various cellular functions. For example, SP1 (Specificity Protein 1) is expressed in many cell types, including healthy and diseased, and regulates expression of genes involved in diverse biological processes such as regulation of the cell cycle, chromatin remodeling, DNA damage responses, cellular housekeeping, differentiation, apoptosis, and others [29, 30]. The motif analyses raise the possibility that cohesin localization may differ in each of the three conditions due to differential association with specific transcription factors. Further investigation is required to determine how these transcription factors may recruit cohesin to new genomic locations in the absence of WIZ. Taken together, our data suggest WIZ regulates cohesin localization and levels on the genome via a mechanism independent of WAPL and the known WIZ binding partner, G9a.

## Conclusions

In summary, understanding how the three-dimensional organization of the genome is dynamically regulated by the cohesin complex and the consequences for gene expression is of critical importance. The results reported here show that WIZ plays an important role in regulating cohesin, largely independent from WAPL in cohesin unloading. The role of WIZ in the regulation of cohesin localization on chromatin is also distinct from its previously reported role with G9a/GLP in mediating the deposition of H3K9me1 and H3K9me2. By regulating the levels and localization of cohesin, WIZ may contribute to proper cycles of cohesin loading onto DNA, extrusion of DNA loops, and unloading from chromatin that is critical for mediating long-range DNA interactions that regulating gene expression.

## Methods

### Cell culture

Mouse embryonic stem cells (mESCs, v6.5, male) were grown in serum + LIF (leukemia inhibitory factor) standard conditions, as previously described except without a feeder layer of irradiated MEFs [16].

### Flow cytometry

Confluent 10 cm plates of mESCs were treated with 10 μM of EdU and incubated at 37^o^ C for 30 min. Cells were then washed with PBS and trypsinized. After spinning down, cell pellets were washed once with PBS then fixed by resuspending in a solution of 500 μL PBS and 4% PFA (paraformaldehyde) and incubating for 15 min at room temperature. Cells were washed twice with 1 mL 1% BSA in PBS. Cells were then resuspended in 1 mL 1% BSA + 0.5% Triton X-100. Cells were incubated at room temperature for 15 min then pelleted. Cells were suspended in a labeling solution of PBS, 1 mM CuSO_4_, ~ 1 μM AlexaFlour 647 Azide, and 100 mM ascorbic acid. Cells were incubated at room temperature for 30 min, protected from light. 1 mL 1% BSA + 0.5% Triton X-100 was added before pelleting cells. Cells were resuspended in 1% BSA + 0.5% Triton X-100. To one biological replicate, 1 drop FxCycle Violet (Thermo Fisher, R37166) reagent was added to each sample before sorting with Attune Nxt flow cytometer (Life Technologies). For two additional biological replicates, cells were resuspended in a solution of 100 μg/mL RNAse and 1 μg/mL DAPI and incubated at 37 ^o^ C for one hour before samples were sorted on Attune NxT flow cytometer. Cell cycle analysis was performed using FCS Express 7.0 software (De Novo, Glendale, CA). Total number of replicates including biological and technical is *n* = 5 for WT and *n* = 6 for *Wiz*^*del*^ cells.

### Immunoblots

Cells were washed in cold PBS then lysed for 5 min on ice in Kischkel buffer (50 mM Tris pH 8.0, 150 mM NaCl, 5 mM EDTA, 1% Triton X-100, protease inhibitor cocktail (PIC), and 1 μM PMSF). Whole cell extracts were then clarified by centrifugation. Laemmli sample buffer with DTT was added and samples were boiled for 10 min before running on a 4–20% mini-PROTEAN TGX gel (BioRad) and transferring to PVDF membrane. Membranes were blocked in 5% milk in TBS-T for 15 min then probed with the following antibodies: α-Cleaved Caspase 3 (9661, Cell Signaling), α-Caspase 3 (9662, Cell Signaling), α-Phospho-Chk1 (p317; 2344S, Cell Signaling), α-Total Chk1 (clone 2G1D5, 2360, Cell Signaling), α-Phospho-p53 (sc-51,690, Santa Cruz), α-Total p53 (clone 1C12, 2524, Cell Signaling), α-phospho-H2AX (Gamma H2AX; clone JBW301, 05–636, Sigma-Aldrich), α-GAPDH–HRP (clone D16H11, 8884, Cell Signaling), and α-GAPDH (clone 6C5, MAB374, MilliporeSigma). SuperSignal West Pico PLUS (34,577, Thermo Fisher) and Supersignal West Femto Maximum Sensitivity (34,095, Thermo Fisher) chemiluminescent substrates were used to visualize signal on an Amersham Imager 600. An apoptosis positive control sample was generated by transducing mouse primary cortical neurons with a lentivirus expressing full length human MAPT for 7 days before collection in Kischkel buffer. A DNA damage control sample was generated by UV treating mESCs for 2 min in the tissue culture hood. Cells were then returned to the incubator for 4 h before collection in Kischkel buffer. Immunoblot quantification: signal for each protein was first normalized to the respective GAPDH signal to control for loading, then *Wiz*^*del*^ signal was normalized to WT signal to determine relative protein levels. Further normalization was performed on phospho-Chk1 and phospho-p53 signal, to respective total protein signal, to determine whether phospho-protein levels significantly decreased in *Wiz*^*del*^ cells. Data from all replicates were then combined and analyzed. PRISM was used to graph data and perform paired, two-tailed t-test analyses on WT versus *Wiz*^*del*^ samples for each protein.

### ChIP-seq analysis

Publicly available ChIP-seq datasets for 1) RAD21 in WT and WAPL-AID mESCs , 2) SMC3 in WT and *G9a* KO / *G9a/GLP* DKO mESCs [26], and 3) WT and *Wiz*^*del*^ mESCs [16] were downloaded from GEO and processed using a previously published custom script [16]. Replicates were merged as raw fastq files before analysis. For the RAD21 ChIPseq in both *Wiz*^*del*^ and WAPL-AID cells, plus their matched WT samples, two biological replicates were merged for each condition. The WAPL-AID condition samples represent cells treated with auxin for 24 h while the WT samples represent 0 h of treatment. These samples were spike-in normalized using human read counts as previously described [16, 31]. The WT SMC3 ChIPseq data presented here represents pooled single replicates from J1 and TT2 WT mESCs (genome-wide pearson correlation = 0.91), and the *G9a* KO ChIPseq data represents pooled single SMC3 ChIPseq replicates from *G9a* KO and *G9a/GLP* DKO cells (genome-wide pearson correlation = 0.95), since biological replicates were not provided in the original manuscript. It should be noted that there have been no separable roles identified for G9a and GLP. SMC3 WT and *G9a* KO samples were not prepared with a spike-in for normalization, instead, any peaks called in the input conditions were removed from peaks called in the ChIP samples. After peak calling, summits are extended by 50 bp on either side and overlapped with repetitive elements (obtained from the UCSC genome browser) using bedtools intersect. Any peaks overlapping a repetitive element is removed. Therefore, peaks are defined as 100 bp regions around peak summits called by MACS2, that do not overlap a repetitive element.

For cis-regulatory element distribution plots in Figs. 1 and 2, CTCF sites are defined from previously published data [16], DNA loop anchors represent SMC1A ChIA-PET anchors from a previous publication [32], enhancers represent peaks of a combined ChIPseq dataset for master transcription factors OCT4, SOX2, and NANOG [16], and TSS elements were obtained using the UCSC genome browser table browser. In Figs. 1 and 2, overlap of ectopic cohesin peaks from different conditions is defined as falling within 2 kb. Distance plots in Figs. 1 and 2 were generated by measuring the distance from an ectopic cohesin peak in one condition to the nearest ectopic cohesin peak in another condition using bedtools closest. The distribution of peak distances in Figs. 1 and 2, as well as the plots in Fig. 3E and F, were generated using ggplot2 (v 3.3.2). Subtractive heatmap tracks in Figs. 1 and 2 were prepared by subtracting WT ChIP signal from mutant ChIP signal using bigWigCompare (deeptools, v 3.2.0). Heatmaps were generated using deeptools computeMatrix and plotHeatmap (v 3.2.0).

Differential occupancy analysis in Fig. 3 was performed using DiffBind (v 3.0.7). Motif analysis was performed using the MEME-ChIP tool from MEME Suite with differential mode (shared RAD21 peaks between WT and *WIZ*^*del*^ as background) [33]. SP1 ChIP-seq data was previously published and re-analyzed [34, 35].

### Data analysis

Cis-regulatory elements in Figs. 1B, D and 2B were defined by the following metrics; CTCF sites were defined from ChIP-seq data [16]. DNA loop anchors were defined from SMC1A ChIA-PET data [32]. Enhancers were defined by merging ChIP-seq datasets for the transcription factors OCT4, SOX2 and NANOG and calling peaks on the merged data [36]. Promoters are Transcription Start Sites (TSSs) defined by the University of California Santa Cruz genome browser (UCSC).

### ChIP-qPCR

Thirty million wildtype mESCs were crosslinked in 1% formaldehyde (Sigma Aldrich, F1635) for 5 min at room temperature, quenched with 125 mM glycine, then washed twice with cold PBS. Cells were lysed first with 10 mL Lysis Buffer 1 (50 mM HEPES-KOH pH 7.5, 140 mM NaCl, 1 mM EDTA, 10% glycerol, 0.5% NP-40, 0.25% Triton X-100, PIC, and PMSF) by incubating cells in the buffer for 10 min at 4C. Nuclei were next lysed with 5 mL Lysis Buffer 2 (10 mM Tris-HCl pH 8, 200 mM NaCl, 1 mM EDTA, 0.5 mM EGTA, PIC, and PMSF) by incubating nuclei in the buffer for 20 min at 4C. Finally, nuclear extracts were washed and resuspended in 1 mL Shearing Buffer (10 mM Tris-HCl pH 7.5, 1 mM EDTA, 0.1% SDS, PIC, and PMSF). Sonication of nuclei was performed on a Covaris E220 with the following settings: Duty Factor 5, PIP/W 140, and 200 cycles per burst for 20 min, to yield a mean chromatin size of ~ 350 bp. After sonication, debris was cleared by centrifugation, and sheared chromatin diluted in Sonication Buffer (20 mM Tris-HCl pH 8, 150 mM NaCl, 2 mM EDTA, 0.1% SDS, 1% Triton X-100, PIC, and PMSF). Immunoprecipitation reactions were performed with either 5μg α-SP1 (07–645, EMD Millipore) or 5μg IgG (P120–101, Bethyl) antibody, as indicated. Immune complexes were recovered on Protein A Dynabeads (Thermo Fisher Scientific, 10002D), which were pre-conjugated with antibodies for 6 h at 4C. Unbound antibody was removed by washing beads three times with PBS before sonicated chromatin was added to antibody-conjugated beads and incubated overnight at 4C. Beads were then washed sequentially with Sonication Buffer, Wash Buffer 1 (20 mM Tris-HCl pH 8, 500 mM NaCl, 2 mM EDTA, 0.1% SDS, 1% Triton X-100, PIC, and PMSF), Wash Buffer 2 (10 mM Tris-HCl pH 8, 250 mM LiCl, 1 mM EDTA, 1% NP-40, PIC, and PMSF), then Wash Buffer 3 (10 mM Tris pH 8, 1 mM EDTA, 50 mM NaCl, PIC, and PMSF). Chromatin was eluted from beads by adding elution buffer (50 mM Tris pH 8, 10 mM EDTA, and 1% SDS) and incubating at 65C for 1 h, spinning down the mixture, then moving the supernatant to a new tube. Proteinase K (20 mg/mL, Thermo Fisher 25–530-049) was added to the immunoprecipitated and input samples and incubated at 65C overnight to reverse crosslinks. ChIP-DNA was cleaned and concentrated using the Zymo ChIP DNA kit (D5205). qPCR was performed using Applied Biosystems PowerUp SYBR Green Master Mix (A25742) on an Applied Biosystems QuantStudio 6 qPCR machine using the following primers: a) Ectopic site 1, FWD: 5′-AAACTTGATTCCATCAGTTCCTC-3′ and REV: 5′-AGAGGAGGAAGAACATGGC-3′, b) Ectopic site 2, FWD: 5′-CTCTGTCCTGCTCTTGGCTC-3′ and REV: 5′-CCTTGGTCCTCACTCCAACC-3′, c) Ectopic site 3, FWD: 5′-GCCTCACATTCCCAACAGGA-3′ and REV: 5′-TTTCTCCGCAAGTTTTCCGC-3′, and d) Control (−SP1), FWD: 5′-ACAGAGCGATACGGCTCAGCAA-3′ and REV: 5′-AAGTGGTAGCCGAAGGCAAGTGAA-3′. ChIP signals were calculated as percent input and ChIP experiments were performed in triplicate. PRISM was used to graph ChIP results.

## Supplementary Information

**Additional file 1.**

**Additional file 2.**

**Additional file 3.**

## Data Availability

The datasets analyzed during the current study are available in the Gene Expression Omnibus repository: GSE137285, GSE44286, GSE98063, GSE116603, GSE107406, GSE31039, GSE54412, GSE73004, GSE116603, GSE135180, GSE138102, GSE126496, GSE56839, GSE57911 (Table S1) [16, 20, 26, 32, 35–40].

## Abbreviations

ChIP-seq: Chromatin immunoprecipitation followed by sequencing
kb: Kilobase
CTCF: CCCTC-binding factor
SMC: Structural maintenance of Chromosomes
SMC1/3: Constitutes the core subunits of cohesin complexes
WIZ: Widely interspaced zinc fingers protein
NIPBL: Nipped b-like
WAPL: Wings apart-like
WT: Wildtype
AID: Auxin-induced degron
KO: Knockout
mESCs: Mouse embryonic stem cells
