# Supplementary material for: Chromosomal localization of cohesin is differentially regulated by WIZ, WAPL, and G9a
Source: BMC Genomics. 2022 Apr 30;23:337. doi: 10.1186/s12864-022-08574-w (PMC9063240; doi:10.1186/s12864-022-08574-w)
Supplement: Supplementary file 2 — Additional file 2. [file 12864_2022_8574_MOESM2_ESM.docx]

**Chromosomal localization of cohesin is differentially regulated by WIZ, WAPL, and G9a**

Megan Justice^1,2^, Audra F. Bryan^1^, Juanita C. Limas^3^, Jeanette Gowen Cook^2,3,4,5^, and Jill M. Dowen^1,2,4,5,6*^

^1^ Integrative Program for Biological and Genome Sciences, University of North Carolina, Chapel Hill, NC, USA

^2^ Curriculum in Genetics and Molecular Biology, University of North Carolina, Chapel Hill, NC, USA

^3^ Department of Pharmacology, University of North Carolina, Chapel Hill, NC, USA

^4^ Department of Biochemistry and Biophysics, University of North Carolina, Chapel Hill, NC, USA

^5^ Lineberger Comprehensive Cancer Center, University of North Carolina at Chapel Hill, Chapel Hill, NC, USA

^6^ Department of Biology, University of North Carolina, Chapel Hill, NC, USA

^*^ Corresponding author (jilldowen@unc.edu)

**Supplemental Figures**


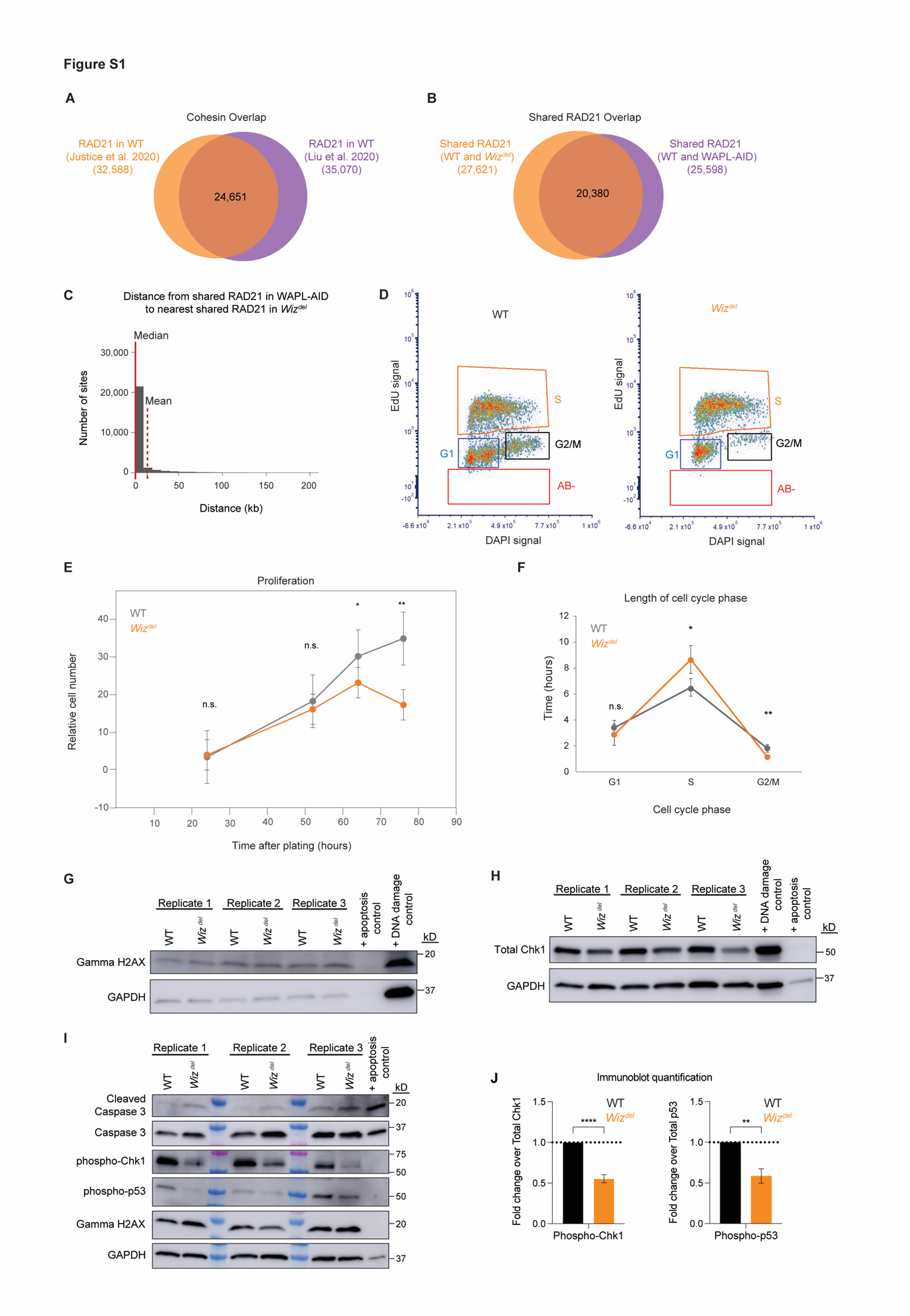


**Figure S1. Overlap of RAD21 peaks in WAPL-AID and *Wiz^del^* cells, related to Figure 1.** (**A**) Overlap of RAD21 peaks in WT cells from Justice et al. 2020 and Liu et al. 2020. (**B**) Overlap of RAD21 peaks shared by WT and *Wiz^del^* cells with RAD21 peaks shared by WT and WAPL-AID cells. (**C**) Histogram showing the distance from a shared RAD21 peak in WAPL-AID cells to the nearest shared RAD21 peak in *Wiz^del^* cells. Median (red solid line) and mean (red dashed line) of the distribution are indicated. (**D**) Scatterplots showing flow cytometry results for WT and *Wiz^del^* cells. Cells in G1 phase are indicated with a blue rectangle. Cells in S phase are indicated with an orange rectangle. Cells in G2/M are indicated with a black rectangle. A negative control, generated with samples lacking antibody staining is indicated with a red rectangle. (**E**) Line plot showing the proliferation rate of WT (grey) and *Wiz^del^* (orange) cells. Significance was tested using an unpaired t-test. Asterisks represent p<0.05 (*) and p<0.001 (**). Error bars represent the standard deviation of the relative cell number of four replicates (biological and technical). For population doubling time calculation, data from the 52- and 64-hour timepoints were used. (**F**) Line plot showing the length of time (in hours) of each cell cycle phase in WT and *Wiz^del^* cells. Lengths were calculated using percent of cells in each phase analyzed by flow cytometry and population doubling time data from panel E. Significance was tested using an unpaired t-test. Asterisks represent p<0.05 (*) and p<0.001 (**). Error bars represent the average deviation of the cell cycle length of 5 (WT) or 6 (*Wiz^del^*) replicates (biological and technical). (**G**) Immunoblot for the DNA damage marker γ-H2AX in WT and *Wiz^del^* whole cell extracts. Positive controls for DNA damage and apoptosis were generated as described in Methods. GAPDH serves as a loading control. This blot is an additional analysis of the same samples shown in Figure 1I and its quantification was combined with Figure 1I quantification to generate Figure 1K. (**H**) Immunoblot for indicator of DNA damage in WT and *Wiz^del^* whole cell extracts. GAPDH serves as a loading control. This blot is an additional analysis of the same samples shown in Figure 1I and its quantification was combined with Figure 1I quantification to generate Figures 1K and S1J. (**I**) Immunoblots for indicators of apoptosis and DNA damage response in WT and *Wiz^del^* whole cell extracts. GAPDH serves as a loading control. These blots represent additional analyses of the same samples shown in Figure 1I and 1J and their quantification was combined with Figure 1I and 1J quantification to generate Figures 1K and S1J. (**J**) Further quantification and normalization of immunoblots in Figure 1I, 1K and S1H-I. Data are presented as mean ±SEM. Phospho-Chk1 signal is normalized to Total Chk1 signal and Phospho-p53 signal is normalized to Total p53 signal. Unpaired, two-tailed t-tests were performed to determine the statistical significance of differences in protein levels between WT and *Wiz^del^* cells. *P < 0.05, **P < 0.01, ***P < 0.001, ****P < 0.0001.

**
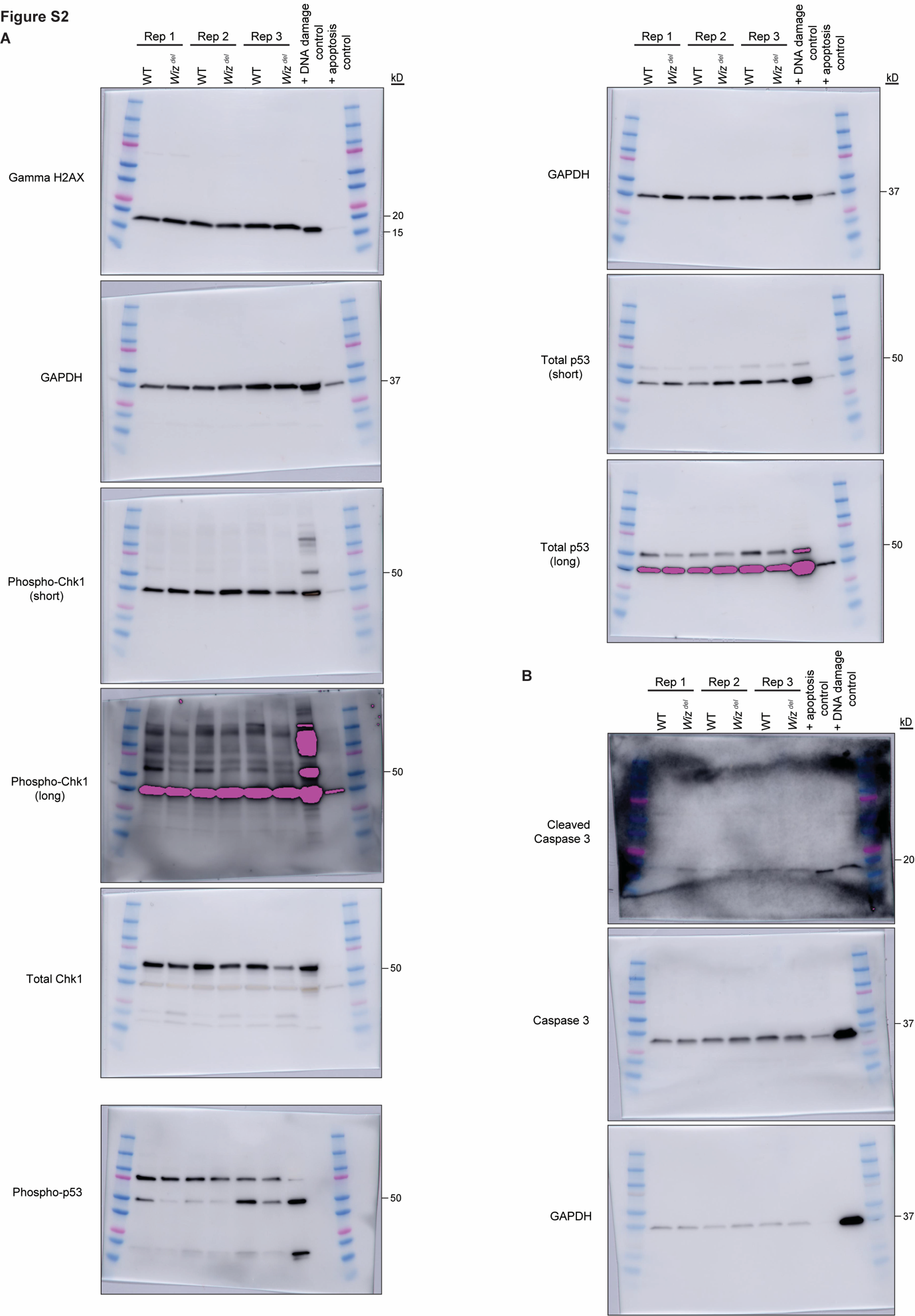
**

**Figure S2. Full-length immunoblots, related to Figure 1.** (**A**) Full length immunoblots for those in Figure 1I, with indicators of apoptosis and the DNA damage response in WT and *Wiz^del^* whole cell extracts. GAPDH serves as a loading control for each membrane. Blots are shown in the order in which they were probed. Gamma H2AX, GAPDH, Phospho-Chk1, then Total Chk1 were probed on one membrane. Phospho-p53, GAPDH, then Total p53 were probed on a second membrane. Short and long exposures for Phospho-Chk1 and Total p53 are included. (**B**) Full length immunoblots for those in Figure 1J, with indicators of apoptosis and the DNA damage response in WT and *Wiz^del^* whole cell extracts. GAPDH serves as a loading control. Blots are shown in the order in which they were probed.


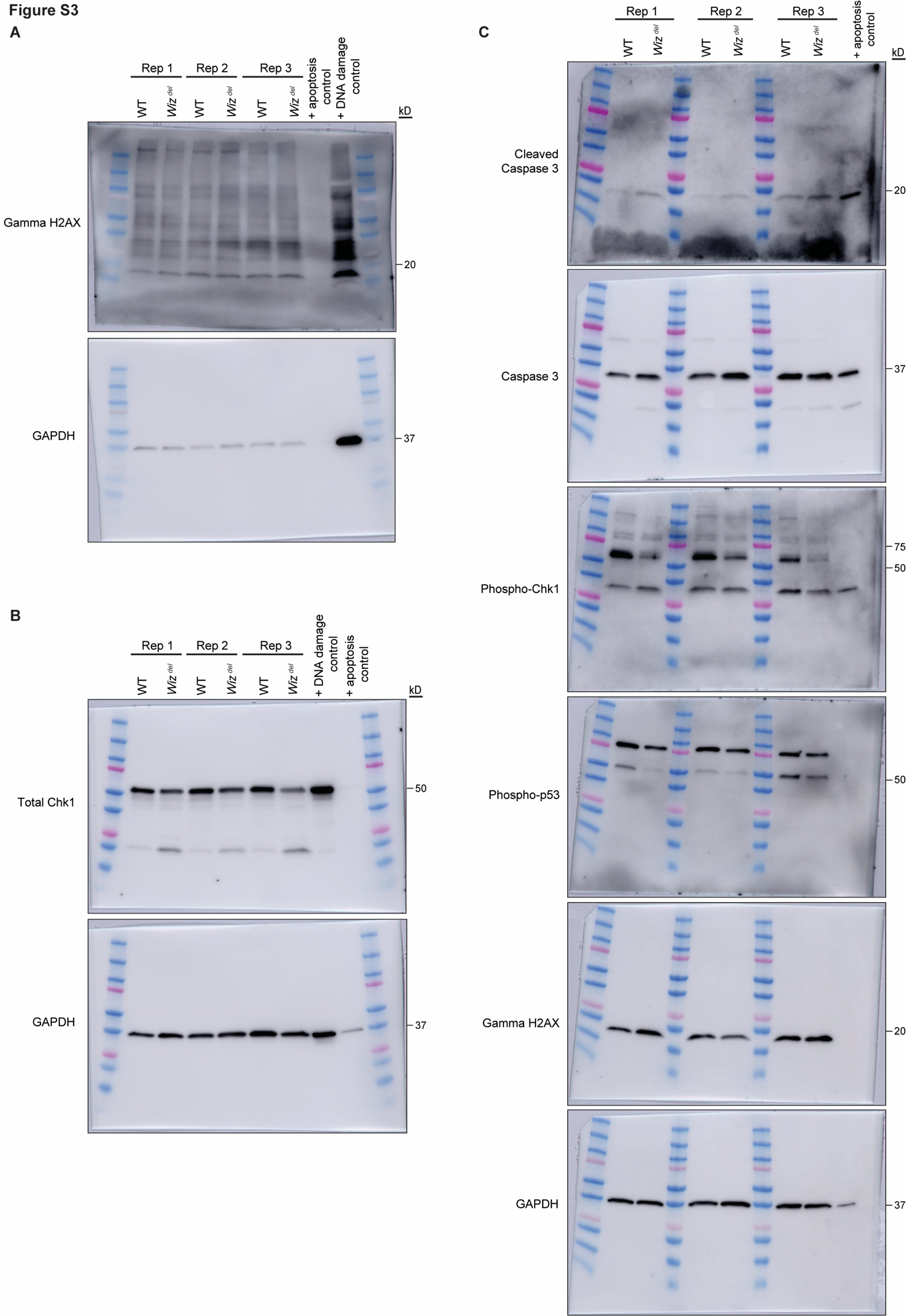


**Figure S3. Full-length immunoblots, related to Figure S1.** (**A**) Full length immunoblots for those in Figure S1G, with indicators of apoptosis and DNA damage response in WT and *Wiz^del^* whole cell extracts. GAPDH serves as a loading control. Blots are shown in the order in which they were probed. (**B**) Full length immunoblots for those in Figure S1H, with indicators of apoptosis and the DNA damage response in WT and *Wiz^del^* whole cell extracts. GAPDH serves as a loading control. Blots are shown in the order in which they were probed. (**C**) Full length immunoblots for those in Figure S1I, with an indicator of apoptosis in WT and *Wiz^del^* whole cell extracts. GAPDH serves as a loading control. Blots are shown in the order in which they were probed.


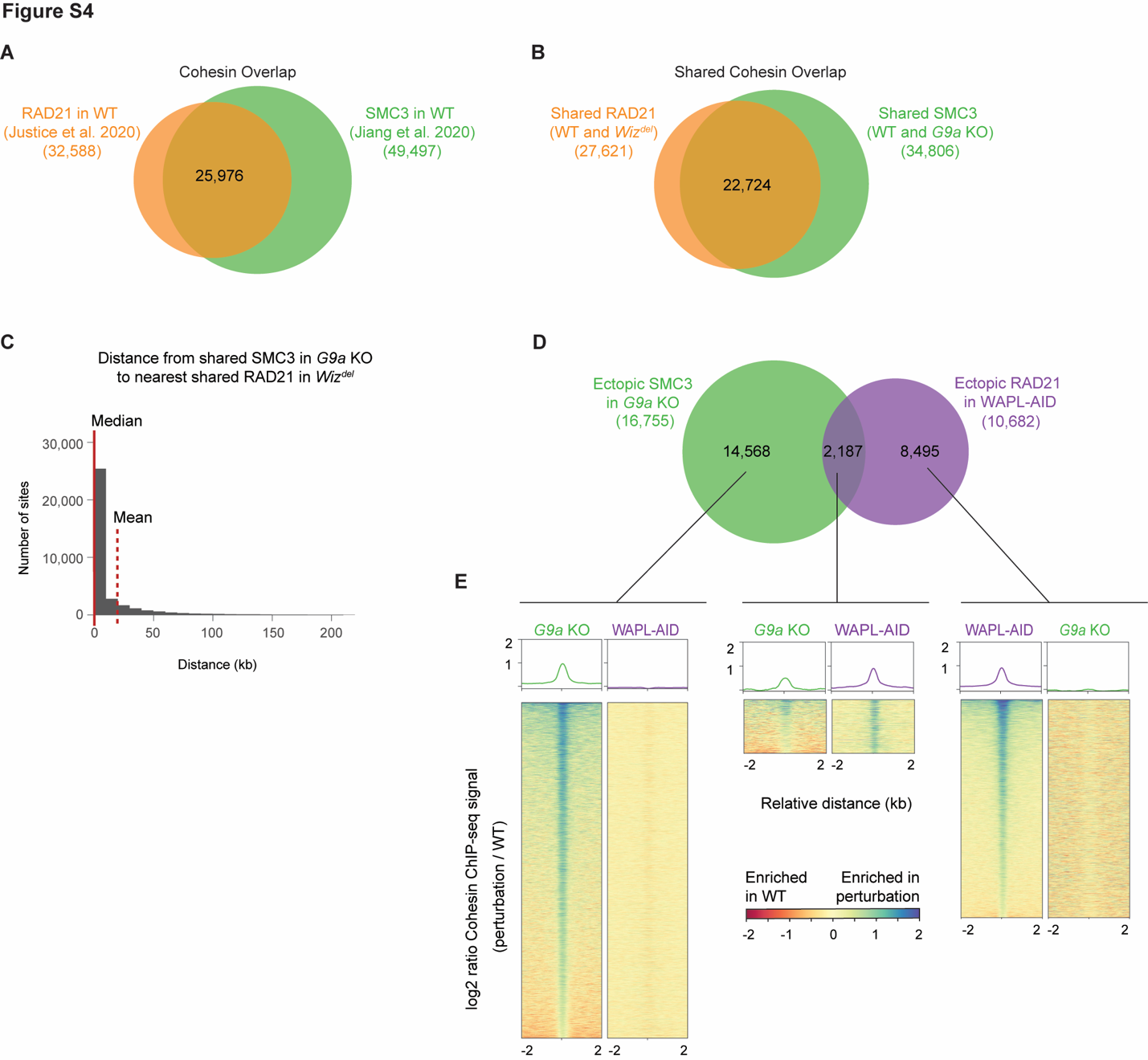


**Figure S4. Overlap of RAD21 peaks in *G9a* KO and *Wiz^del^* cells, related to Figure 2.** (**A**) Overlap of WT RAD21 peaks from Justice et al. 2020 and WT SMC3 peaks from Jiang et al. 2020. (**B**) Overlap of RAD21 peaks shared by WT and *Wiz^del^* cells with SMC3 peaks shared by WT and G9a KO cells. (**C**) Histogram showing the distance from each shared SMC3 peak in *G9a* KO cells to the nearest shared RAD21 peak in *Wiz^del^* cells. Median (red solid line) and mean (red dashed line) of the distribution are indicated. (**D**) Overlap of ectopic SMC3 peaks in *G9a* KO cells with ectopic RAD21 peaks in WAPL-AID cells. (**E**) Heatmaps showing the log2 ratio of cohesin signal (perturbation / WT) at ectopic cohesin peaks identified in *G9a* KO cells only (left heatmaps, sorted from highest to lowest signal in *G9a* KO column), peaks identified as ectopic in both *G9a* KO cells and WAPL-AID (center heatmaps, sorted from highest to lowest signal in *G9a* KO column), and peaks identified only in WAPL-AID cells (right heatmaps, sorted from highest to lowest signal in WAPL-AID column).


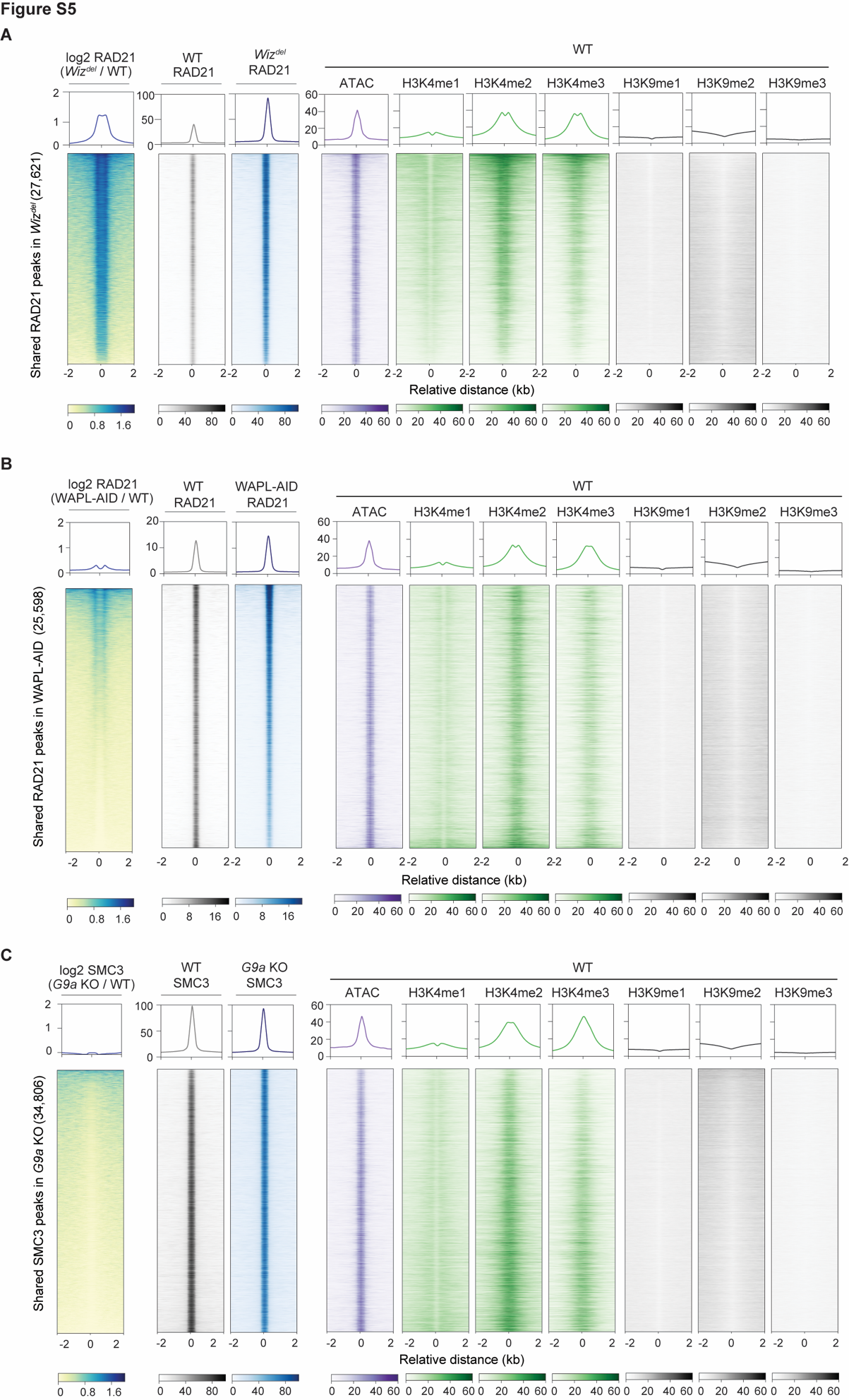


**Figure S5. Shared cohesin peaks in *Wiz^del^*, WAPL-AID, and *G9a* KO cells display similar chromatin landscapes, related to Figure 4.** (**A**) Heatmaps showing RAD21 ChIP-seq signal (log2 (*Wiz^del^* / WT)), WT RAD21 ChIP-seq signal, *Wiz^del^* RAD21 ChIP-seq signal, WT ATAC-seq signal, and WT ChIP-seq signal for H3K4me1, H3K4me2, H3K4me3, H3K9me1, H3K9me2, and H3K9me3 at shared RAD21 peaks detected in *Wiz^del^* cells. (**B**) Heatmaps showing RAD21 ChIP-seq signal (log2 (WAPL-AID / WT)), WT RAD21 ChIP-seq signal, WAPL-AID RAD21 ChIP-seq signal, WT ATAC-seq signal, and WT ChIP-seq signal for H3K4me1, H3K4me2, H3K4me3, H3K9me1, H3K9me2, and H3K9me3 at shared RAD21 peaks detected in WAPL-AID cells. (**C**) Heatmaps showing SMC3 ChIP-seq signal (log2 (*G9a* KO / WT)), WT RAD21 ChIP-seq signal, *G9a* KO SMC3 ChIP-seq signal, WT ATAC-seq signal, and WT ChIP-seq signal for H3K4me1, H3K4me2, H3K4me3, H3K9me1, H3K9me2, and H3K9me3 at shared SMC3 peaks detected in *G9a* KO cells.

**Supplemental Tables**

**Table S1. Datasets used in the current study. (Excel file Table S1.xls)**

**Table S2. Motif Analysis, related to Figure 5. (Excel file Table S2.xls)**
